# Health Under Haze: Symptom Burden Among Adult Outpatients During the 2024‐2025 Winter Smog Season in Lahore, Pakistan

**DOI:** 10.1002/puh2.70349

**Published:** 2026-08-17

**Authors:** Mirza Ameer Faizan Ali, Bushra Iftikhar, Syed Zeeshan Haider, Aqsa Ikram, Ayesha Masood, Ghaniya Ali, Eunice Damilola Wilkie, Jude Oluwapelumi Alao

**Affiliations:** ^1^ Pathology Department, Azra Naheed Medical College Superior University Lahore Pakistan; ^2^ Institute of Molecular Biology and Biotechnology The University of Lahore Lahore Pakistan; ^3^ Biochemistry Department, Azra Naheed Medical College Superior University Lahore Pakistan; ^4^ Pathology Department University College of Medicine and Dentistry, The University of Lahore Lahore Pakistan; ^5^ Pathology Department Al‐Aleem Medical College, The University of Health Sciences Lahore Pakistan; ^6^ Microbiology Department Adeleke University Ede, Osun State Nigeria; ^7^ School of Science Auckland University of Technology Auckland New Zealand

**Keywords:** air pollution, air quality, environmental exposure, particulate matter, respiratory tract diseases, urban health

## Abstract

**Background:**

Severe urban air pollution is a major public health challenge in many low‐ and middle‐income countries. Lahore experiences recurrent winter smog episodes with pollution levels far exceeding international guidelines, but hospital‐based evidence on associated symptom burden remains limited.

**Methods:**

We conducted a cross‐sectional study of 400 adult outpatients attending a tertiary hospital in Lahore between October 2024 and January 2025, during a period of sustained severe air pollution. A structured questionnaire collected data on demographic characteristics, behavioural factors, occupational exposure proxies, medical history and respiratory and non‐respiratory symptoms. Symptom burden was analysed using descriptive statistics and multivariable regression models, including an exploratory composite vulnerability index. City‐level air quality index (AQI), PM_2.5_, PM_10_, carbon monoxide (CO), and temperature data were summarised relative to World Health Organization guidelines to contextualise environmental conditions.

**Results:**

In this health‐seeking outpatient sample, 98% of participants reported at least one symptom. The most common complaints were shortness of breath (74%), eye irritation (56%), cough (53%) and sore throat (50%). PM_2.5_ and PM_10_ exceeded WHO 24‐h guideline values on all monitored days. Mean AQI was 180, mean PM_2.5_ was 96 µg/m^3^, and mean CO was 979, indicating sustained multi‐pollutant exposure. Higher cumulative vulnerability was associated with greater symptom counts (*p* < 0.01), and current smoking was associated with increased symptom burden (incidence rate ratio [IRR] 1.15, 95% CI 1.00‐1.32). Routine mask use was not significantly associated with lower symptom burden (IRR 0.96, 95% CI 0.84‐1.09). Perceived worsening air quality showed limited concordance with vulnerability and symptom patterns.

**Conclusion:**

During Lahore's 2024–2025 winter smog season, adult outpatients reported substantial multisystem symptom burden. These findings support strengthened environmental control measures and public health responses in heavily polluted urban settings.

## Graphical abstract

Adult outpatients in Lahore reported substantial respiratory and non‐respiratory symptom burden during the severe 2024‐2025 winter smog season. City‐level monitoring showed persistent PM_2.5_ and PM_10_ exceedances, supporting the need for population‐level air‐quality controls alongside individual protective measures.

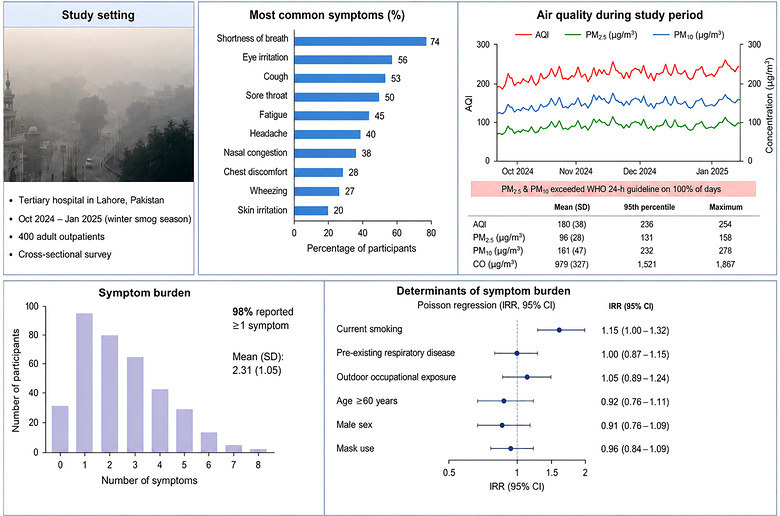

## 1 Introduction

Ambient air pollution is a major global health concern and a leading environmental risk factor for morbidity and mortality, with fine particulate matter exposure contributing to millions of deaths annually worldwide [1]. In South Asia, densely populated urban centres experience recurrent winter smog episodes shaped by atmospheric stagnation, combustion emissions and regional agricultural burning [2, 3]. In Lahore, long‐term monitoring has documented persistently extreme aerodynamic diameter ≤2.5 µm (PM_2.5_) concentrations, indicating that seasonal smog occurs against a backdrop of chronic urban air pollution rather than as isolated events [4]. During late 2024, Lahore was widely reported among the world's most polluted cities, with air quality index (AQI) values exceeding 300 and PM_2.5_ levels far above World Health Organization guideline thresholds [5, 6].

Winter smog in Lahore is therefore not only an atmospheric phenomenon but also a manifestation of interacting social‐ecological processes, including urbanisation, transport and energy systems, land‐use patterns and meteorological conditions that trap pollutants. Recent climate signals, including record global warmth reported for 2024, may further influence the frequency and persistence of severe pollution episodes through effects on atmospheric dynamics and dispersion [7]. In such settings, where exposure can be sustained and widespread, characterising symptom burden and vulnerability among affected outpatients may be more informative than attempting to distinguish individual exposure contrasts.

Despite the recognised severity of Lahore's smog, hospital‐based evidence describing symptom burden and vulnerability patterns during periods of extreme pollution remains limited. This study therefore analysed self‐reported respiratory and non‐respiratory symptoms among adult outpatients attending a tertiary care facility in Lahore during the 2024‐2025 smog season. We aimed to characterise symptom prevalence, patterns and heterogeneity and to examine how symptom burden aligned with vulnerability features and behavioural responses, alongside perceived air quality. We also contextualised the study period using city‐level AQI, PM_2.5_, aerodynamic diameter ≤10 µm (PM_10_), carbon monoxide (CO) and temperature data relative to World Health Organization guideline values, to situate reported health experiences within the broader urban environmental conditions.

## 2 Methods

### 2.1 Study Design and Setting

This study employed a cross‐sectional observational design to integrate routine ambient air quality monitoring with an outpatient health assessment during the 2024‐2025 winter smog period in Lahore, Pakistan. Data were collected between October 2024 and January 2025, a timeframe characterised by recurrent and sustained episodes of severe air pollution. The primary objective was to characterise city‐level ambient pollution conditions using routine environmental monitoring data and to assess corresponding patterns of self‐reported respiratory and non‐respiratory symptoms, perceived air quality and vulnerability profiles among adult outpatients, rather than to attribute individual health outcomes to specific pollutant concentrations.

Health data were collected at the outpatient department (OPD) of Gulab Devi Teaching Hospital, Lahore (31.483304811761304, 74.3427590669488), a central tertiary care facility serving patients from a wide range of residential and socioeconomic backgrounds across the metropolitan area. This setting enabled the capture of health information reflective of routine healthcare utilisation under conditions of heightened environmental stress. Given the persistence, spatial pervasiveness and severity of ambient air pollution during the study period, the analysis was designed to document symptom burden, symptom distributions and heterogeneity within this outpatient sample under conditions of near‐universal exposure, while using environmental monitoring data to contextualise exposure intensity and persistence at the city scale.

### 2.2 Study Sample and Recruitment

The study sample comprised adult patients aged 18 years and above attending the OPD during the study period. Eligibility was restricted to individuals who had resided in Lahore for a sufficient period to plausibly experience ambient smog exposure, who were able to provide informed consent and complete the study questionnaire, and who presented to the clinic specifically because of perceived worsening health during the smog season. Recruitment was designed to capture participants living in areas with varying levels of ambient air pollution, as indicated by city‐level environmental monitoring data, and to include both individuals with pre‐existing respiratory or cardiovascular conditions and those without known chronic disease. This approach enabled comparison across vulnerability strata and supported assessment of differential symptom burden under conditions of sustained winter smog.

Because eligibility required presentation to the outpatient clinic for perceived worsening health during the smog season, the cohort was interpreted as a health‐seeking outpatient sample enriched for symptomatic or concerned individuals, rather than as a representative sample of Lahore residents.

The sample size was determined a priori using the standard single‐proportion formula for cross‐sectional studies. Assuming a conservative expected prevalence of smog‐associated health symptoms of 50%, a 95% confidence level, and a margin of error of 5%, the minimum required sample size was calculated to be 384 participants. To account for potential non‐response, incomplete questionnaires and exclusions during quality control, the target sample size was increased to 400 participants, who were ultimately enrolled. This sample size was considered sufficient to provide precise prevalence estimates and to support the planned multivariable analyses examining vulnerability factors, behavioural modifiers and symptom burden during the smog season.

### 2.3 Exclusion Criteria

Participants were excluded from the study if they had chronic medical conditions unlikely to be influenced by air pollution and therefore expected to confound symptom reporting, such as advanced systemic illnesses unrelated to environmental exposure. Individuals with a diagnosed history of ischaemic heart disease or other severe cardiac conditions were also excluded, as these conditions could independently account for acute symptom presentation. Participants were further excluded if they had known sinobronchial syndrome or other chronic respiratory disorders not attributable to PM_2.5_ pollution, or if they were experiencing a concurrent acute illness at the time of recruitment. Current use of prescription medication for smoking cessation constituted an additional exclusion criterion because of its potential to confound respiratory symptom reporting. Pregnant women were excluded due to their distinct physiological vulnerability and pregnancy‐related respiratory changes, which differ from those of the general adult outpatients. Individuals who were unable to fully participate in the questionnaire due to cognitive or communication limitations were also excluded.

### 2.4 Data Collection and Questionnaire Measures

Data was collected using a structured, interviewer‐administered questionnaire designed to systematically capture demographic characteristics, behavioural factors, exposure proxies, medical history and symptom profiles relevant to ambient smog exposure (Supporting Information File 1). An interviewer‐administered format was employed to minimise missing data, ensure consistent interpretation of questions and reduce misclassification arising from literacy or comprehension differences among participants.

#### 2.4.1 Demographic and Behavioural Variables

Demographic and behavioural variables collected included age and sex, smoking status, occupational category, performance of regular outdoor work, typical duration of daily outdoor exposure, use of personal protective measures such as face masks, and self‐reported stress levels during the smog period. These variables were selected because they are well‐established determinants of both exposure intensity and susceptibility to air pollution‐related health effects. Occupational category and outdoor activity were used to approximate relative exposure to ambient air pollution, whereas smoking status and stress were included as potential confounders and effect modifiers of respiratory and systemic symptoms. The inclusion of mask use allowed assessment of behavioural practices that may influence symptom experience without presuming protective efficacy.

#### 2.4.2 Medical History and Symptom Assessment

Participants reported pre‐existing respiratory and cardiovascular conditions to identify individuals with heightened biological susceptibility to air pollution. Symptom assessment focused on both respiratory manifestations, including persistent cough, shortness of breath, sore throat and chest discomfort, and non‐respiratory symptoms such as eye irritation, fatigue and skin irritation. Symptoms were recorded as binary indicators to standardise reporting and to reduce recall ambiguity. In addition to individual symptom variables, a composite symptom count was derived to reflect overall symptom burden, thereby enabling analyses that captured cumulative health impact rather than isolated symptom presence.

#### 2.4.3 Perceived Air Quality

Participants were asked whether they perceived worsening air quality during the smog period. This measure was included to assess the relationship between subjective environmental perception, objective vulnerability markers and reported health outcomes. Inclusion of perceived air quality enabled examination of perception–symptom alignment and discordance, which is increasingly recognised as an essential dimension of environmental health risk communication and public response during pollution episodes. Efforts were made during participant recruitment to ensure representation across age groups, sexes and residential areas in Lahore to capture variability in exposure contexts and lived experiences during the smog season.

### 2.5 Environmental Air Quality and Temperature Data

Ambient air quality data were obtained from publicly available city‐level environmental monitoring records for Lahore, Pakistan, using the AQI.in platform. This platform aggregates measurements from a network of fixed monitoring stations and is widely used for urban air quality surveillance in South Asia, providing temporally continuous indicators suitable for characterising city‐level ambient conditions. For the purposes of environmental assessment, we extracted the United States AQI, PM_2.5_, PM_10_ and CO as primary indicators of ambient pollution during the smog season.

The selection of these parameters was guided by their relevance to both air quality monitoring practice and pollution risk assessment. PM_2.5_ was prioritised as a key indicator of fine particulate pollution with well‐established links to adverse respiratory and cardiovascular outcomes and its central role in international air quality standards [8]. PM_10_ was included to capture coarser inhalable particles associated with dust resuspension, traffic emissions and biomass burning, which are prominent features of Lahore's winter pollution profile [9]. CO was used as a marker of incomplete combustion from vehicular traffic and other combustion sources that contribute to urban air pollution loads [10]. The composite AQI was retained as an integrative metric reflecting overall air quality conditions and public‐facing risk communication during the study period.

Other pollutants available on the monitoring platform (e.g., ozone or nitrogen dioxide) were not included because they were either inconsistently reported across the study period or less central to the particulate‐dominated winter smog regime in Lahore. This targeted selection was intended to optimise the relevance, interpretability and consistency of the monitoring dataset for environmental assessment.

Daily temperature data were obtained from nearby meteorological stations using the Meteostat framework. Data were extracted from 1 November 2024 to 31 January 2025, corresponding to the pre‐smog and peak smog season. Using the geographic coordinates of Lahore (31.5204° N, 74.3587° E), the six closest weather stations were identified, and daily mean, minimum and maximum temperatures were averaged across these stations. Temperature was included as an environmental modifier of pollutant dispersion, atmospheric stability and smog persistence, thereby strengthening the contextual assessment of ambient conditions.

All environmental data were processed at the daily level and summarised descriptively to characterise temporal patterns of air quality during the study period. Pollutant concentrations were evaluated against the WHO 2021 Air Quality Guidelines to quantify the frequency, magnitude and persistence of exceedances [9]. PM_2.5_ and PM_10_ were compared with their respective 24‐h guideline values to assess the intensity and temporal variability of ambient pollution at the city level.

These environmental datasets were used to provide a city‐level assessment of ambient conditions rather than to estimate individual‐level exposures. Because precise dates of participants’ symptom onset were not available, direct linkage between individual respondents and specific daily pollutant values was not undertaken to avoid substantial exposure misclassification. Accordingly, environmental analyses served to contextualise the monitoring record alongside health data, enabling a synthesis of environmental conditions and symptom patterns in the outpatient sample during the smog season.

### 2.6 Data Management and Variable Construction

All participant data were anonymized and assigned unique study identifiers to ensure confidentiality and reproducibility of analyses. Questionnaire responses were checked for completeness and internal consistency, and variables were coded using standardised, pre‐specified rules to support transparent and replicable assessment. Binary variables were constructed in accordance with the original questionnaire response options. Composite indicators for any respiratory and any non‐respiratory symptoms were created to facilitate parsimonious analysis of health outcomes alongside environmental monitoring data. A total symptom count variable was generated by summing individual symptom items, enabling quantitative assessment of overall symptom burden in relation to ambient pollution conditions.

An ‘outdoor worker’ status variable was derived from occupational category and reported time spent outdoors as a pragmatic exposure proxy in the absence of personal air pollution monitoring. This approach aligns with common practice in environmental exposure assessment where direct individual‐level measurements are unavailable. All derived variables were defined prior to inferential analyses to minimise data‐driven specification and enhance transparency. Original and derived variables were analysed in parallel to allow complementary assessment of symptom‐specific outcomes and aggregate health burden, supporting a balanced synthesis of monitoring data and outpatient symptom patterns during the smog season.

### 2.7 Statistical Analysis

All statistical analyses were conducted using R statistical software (Version 2026.01.0+392). Statistical inference was based on two‐sided tests with an alpha level of 0.05. In line with contemporary epidemiological reporting standards, emphasis was placed on effect sizes and 95% confidence intervals rather than on *p* values alone, particularly given the exploratory nature of several analyses and the potential for imprecision in self‐reported exposure proxies.

#### 2.7.1 Descriptive Analysis

Participant demographic characteristics, exposure proxies, behavioural factors and health outcomes were summarised using frequencies and percentages for categorical variables, and means with standard deviations or medians with interquartile ranges for continuous variables, as appropriate. Symptom prevalence was calculated for each reported symptom and for composite endpoints, including any symptom, respiratory symptoms and non‐respiratory symptoms. These descriptive analyses were conducted to establish the baseline exposure profile, vulnerability distribution and overall symptom burden within the outpatient sample prior to inferential modelling.

#### 2.7.2 Vulnerability and Susceptibility Analysis

Multivariable logistic regression models were fitted to examine associations among key exposure proxies, susceptibility factors and respiratory symptoms. Exposure and vulnerability variables included occupational outdoor exposure, smoking status, pre‐existing respiratory disease, age group, sex and mask use. Adjusted odds ratios (aORs) with 95% confidence intervals were reported. Multicollinearity was assessed using variance inflation factors to ensure model stability. This analysis was undertaken to identify outpatient subgroups experiencing disproportionate respiratory symptom burden during the smog season, while accounting for multiple overlapping vulnerability factors.

#### 2.7.3 Behavioural Protection and Effect Modification Analysis

To evaluate whether protective behaviours modified associations between exposure and health outcomes, interaction models were fitted using multivariable logistic regression. Specifically, interaction terms between outdoor occupational exposure and mask use were included to assess effect modification rather than causal prevention. These analyses were interpreted cautiously, focusing on whether symptom‐exposure relationships differed by behavioural context rather than inferring protective efficacy. Predicted probabilities with confidence intervals were generated to aid the interpretation of interaction effects and provide intuitive visual summaries of modelled relationships.

#### 2.7.4 Symptom Burden and Pattern Analysis

Symptom burden was examined using complementary approaches to move beyond binary outcomes. First, total symptom count was analysed using Poisson regression, with formal assessment of overdispersion guiding model selection; negative binomial models were reserved for scenarios where overdispersion was detected. Incidence rate ratios (IRRs) with 95% confidence intervals were reported. Second, unsupervised cluster analysis was conducted using symptom‐level binary variables to identify distinct symptom pattern groups, including low burden, respiratory‐dominant and mixed multisystem profiles. Multinomial logistic regression was subsequently used to explore predictors of cluster membership. These analyses were conducted to characterise heterogeneity in symptom expression and to identify whether exposure or behavioural factors explained observed symptom patterns.

Because any respiratory symptom was highly prevalent and therefore had limited discriminatory value, sensitivity analyses were conducted using stricter respiratory‐health outcomes. These included the presence of two or more respiratory symptoms, respiratory symptom count and a clinically focused composite defined as shortness of breath with persistent cough and/or chest discomfort. These analyses were used to assess whether findings were robust when respiratory burden was defined more stringently.

#### 2.7.5 Composite Vulnerability Index Analysis

An exploratory composite vulnerability score was constructed by summing four pre‐specified indicators: outdoor occupational exposure, current smoking, pre‐existing respiratory disease and age 60 years or older. Each component was assigned equal weight for simplicity and interpretability. The score was not intended to represent a validated vulnerability index or to imply equivalent biological importance of each component. Rather, it was used as a pragmatic descriptive tool to examine whether symptom burden increased with accumulation of common susceptibility and exposure‐related characteristics.

#### 2.7.6 Perceived Air Quality and Perception–Symptom Discordance Analysis

Associations between perceived worsening air quality and objective vulnerability markers were examined using cross‐tabulations and regression models. Penalised logistic regression was employed where necessary to address quasi‐separation arising from highly imbalanced perception responses. Additional models assessed whether perceived air quality independently predicted respiratory symptoms and total symptom burden after adjustment for confounders. To further explore human‐environment interaction, perception‐symptom discordance was visualised using matrix‐based and flow‐based graphics, illustrating alignment and misalignment between subjective environmental perception and reported health burden. These analyses were conducted to assess whether perception captured health risk beyond objective vulnerability proxies.

#### 2.7.7 Environmental Contextualisation and Visual Exposure Assessment

Ambient air quality and meteorological data were summarised descriptively to characterise environmental conditions during the study period. Daily concentrations of key pollutants and temperature metrics were aggregated and compared against the WHO guideline values. Environmental conditions were visualised using time‐series plots, exposure gradient heatmaps, and a calendar‐based exceedance plot that illustrates the persistence and intensity of PM_2.5_ guideline exceedances across the smog season. These analyses were conducted strictly for contextual interpretation and visualisation of exposure conditions and were not used to model individual‐level exposure‐outcome relationships.

## 3 Results

The outpatient sample was predominantly middle‐aged, with a higher proportion of male participants, and represented a broad range of occupational and exposure profiles (Table 1). Approximately half of the participants were categorised as indoor workers, whereas over one‐third were engaged in occupations characterised by high outdoor exposure, and a substantial proportion reported spending more than 5 h outdoors daily. Pre‐existing respiratory conditions and current smoking were common, reflecting a sample with underlying vulnerability during the smog season. Reported use of face masks was comparatively low.

**TABLE 1:** Demographic characteristics, exposure profile and baseline health status of participants.

Variable	Level	Value
Age, mean (SD)		47.3 (16.1)
Sex	Female	154 (38.5%)
	Male	246 (61.5%)
Occupation	Farmer	33 (8.2%)
	Household	139 (34.8%)
	Labour	111 (27.8%)
	Office employee	62 (15.5%)
	Shopkeeper	55 (13.8%)
Occupation exposure group	Indoor	201 (50.2%)
	Mixed	55 (13.8%)
	Outdoor high	144 (36.0%)
Hours outdoors (category)	1‐3 h	123 (30.8%)
	>3 h	75 (18.8%)
	>5 h	202 (50.5%)
Current smoking (Yes)		143 (35.8%)
Pre‐existing respiratory disease (Yes)		165 (41.2%)
Mask use (Yes)		113 (28.2%)

### 3.1 Symptom Prevalence Across Occupational Exposure Strata

**FIGURE 1:**
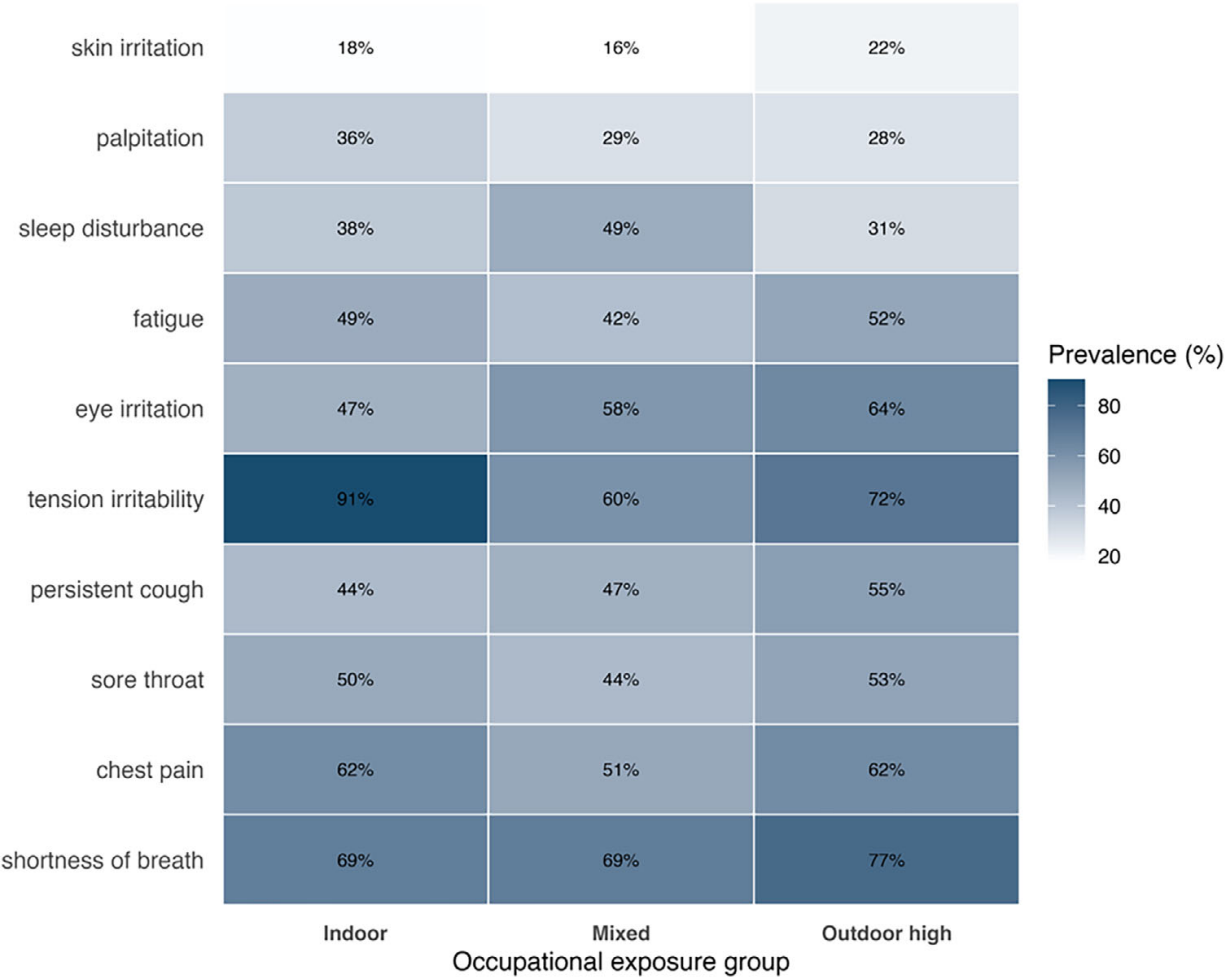
Symptom prevalence across occupational exposure strata.

Analysis of symptom prevalence across occupational exposure groups revealed clear gradients for several key symptoms, with higher prevalence generally observed among participants with greater outdoor exposure (Figure 1). Respiratory symptoms showed the most consistent exposure‐related patterns. Shortness of breath was highly prevalent across all groups but was most pronounced among participants in the outdoor high‐exposure group (77%), compared with the indoor (69%) and mixed‐exposure groups (69%). Persistent cough and sore throat also showed higher prevalence among those with outdoor high exposure (55% and 53%, respectively) than among those in indoor and mixed groups.

Chest pain was common across all exposure strata, with comparable prevalence in indoor and outdoor high groups (both 62%), and slightly lower prevalence in the mixed exposure group (51%), suggesting anelevated burden that was not confined to a single exposure category. Eye irritation exhibited a clearer gradient, increasing from 47% in indoor workers to 58% in the mixed group and 64% among those with outdoor high exposure, consistent with increased ocular irritation in more polluted outdoor environments.

Non‐respiratory symptoms displayed more heterogeneous patterns. Fatigue was prevalent across all exposure groups but was highest among participants with outdoor high exposure (52%), whereas sleep disturbance peaked in the mixed exposure group (49%) before declining in the outdoor high group (31%). Palpitations showed a decreasing trend with increasing outdoor exposure, whereas skin irritation was relatively low across all groups, though slightly higher in the outdoor high exposure category (22%). Notably, tension and irritability were highly prevalent across all groups, particularly among indoor workers (91%).

#### 3.1.1 Composite Vulnerability Index and Symptom Burden

**FIGURE 2:**
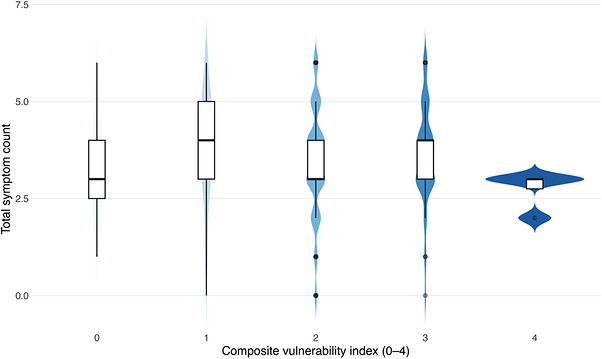
Symptom count by composite vulnerability index.

In exploratory analyses, higher scores on the composite vulnerability measure were associated with higher symptom counts (Figure 2). Participants with lower vulnerability scores exhibited lower median symptom counts and narrower distributions. As the vulnerability index increased, the distribution of total symptom counts shifted upward, with higher medians and greater dispersion, reflecting increased heterogeneity in symptom experience among more vulnerable individuals. Participants in the highest vulnerability strata showed consistently elevated symptom counts, with distributions concentrated at higher values and fewer low‐symptom observations.

#### 3.1.2 Perception‐Symptom Discordance during the Smog Season

**FIGURE 3:**
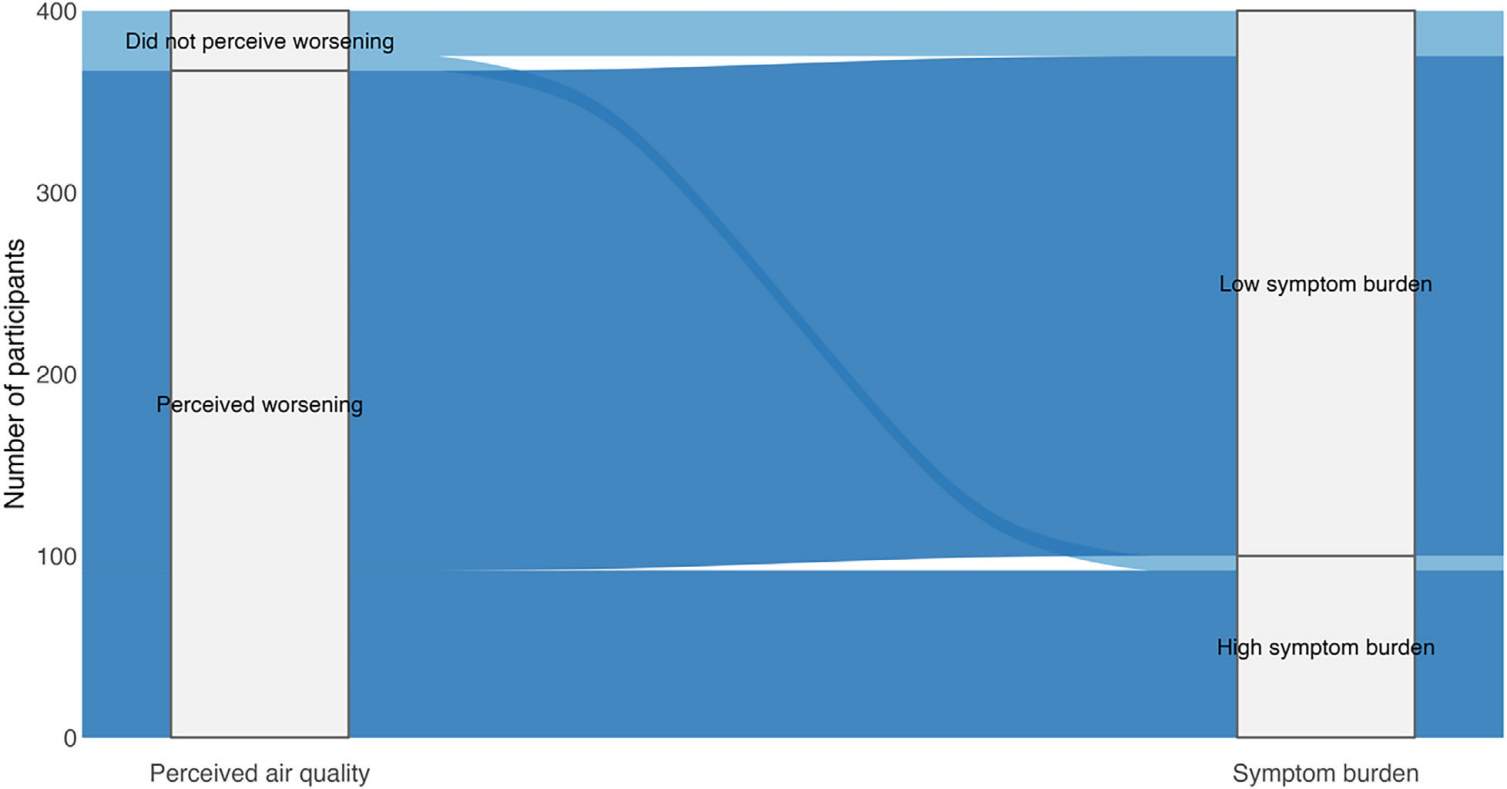
Perception–symptom discordance during the smog season.

The alluvial analysis revealed substantial discordance between participants’ perception of worsening air quality and their reported symptom burden during the smog season (Figure 3). A large proportion of participants who did not perceive a deterioration in air quality nonetheless reported a high symptom burden. Conversely, although many participants who perceived worsening air quality reported symptoms, a considerable fraction of this group remained in the low‐symptom‐burden category.

### 3.2 Model Diagnostics

#### 3.2.1 Primary Model

In the primary multivariable logistic regression analysis assessing factors associated with the presence of any respiratory symptom, no evidence of multicollinearity was observed; variance inflation factors for all covariates remained well below commonly accepted thresholds, indicating stable estimation of model parameters. After adjustment for behavioural and demographic factors, outdoor occupational exposure was not independently associated with respiratory symptom reporting (aOR = 1.33, 95% CI 0.34‐4.59). Current smoking was associated with higher odds of respiratory symptoms (aOR = 2.79), although this association did not reach conventional statistical significance and was accompanied by wide confidence intervals (95% CI 0.90‐9.73), suggesting limited precision. Similarly, participants with pre‐existing respiratory disease had higher odds of reporting symptoms (aOR = 1.94), but this association was not statistically significant (95% CI 0.61‐7.55).

Participants aged 60 years and above had lower odds of reporting respiratory symptoms than younger participants (aOR = 0.39, 95% CI 0.08‐2.24), although this difference was not statistically significant. Mask use was associated with lower odds of respiratory symptoms (aOR = 0.36, 95% CI 0.11‐1.17), indicating a potential protective association; however, the confidence interval included the null value, and the result did not reach statistical significance. In contrast, sex was significantly associated with respiratory symptom reporting, with male participants exhibiting substantially lower odds of reporting symptoms compared with female participants (aOR = 0.05, 95% CI 0.00‐0.30; *p* = 0.006).

#### 3.2.2 Secondary Model

In the secondary analysis examining symptom burden, symptom count was modelled using Poisson regression. Assessment of model dispersion indicated no evidence of overdispersion, with a dispersion ratio of 0.51 and a non‐significant Pearson chi‐squared test (*p* = 1.00). In the Poisson regression analysis examining factors associated with overall symptom burden, perceived worsening of air quality was not significantly associated with symptom count after adjustment for covariates (IRR = 0.95, 95% CI 0.76‐1.18). Outdoor occupational exposure similarly showed no independent association with the number of reported symptoms (IRR = 1.06, 95% CI 0.93‐1.21).

Current smoking was associated with a higher symptom burden, with smokers reporting a greater number of symptoms compared with non‐smokers (IRR = 1.15, 95% CI 1.00‐1.32), indicating a modest but statistically significant increase in symptom count. Pre‐existing respiratory disease was not significantly associated with symptom burden in this model (IRR = 0.92, 95% CI 0.81‐1.03). Neither age nor sex was significantly associated with symptom count, and mask use was not independently associated with overall symptom burden (IRR = 0.96, 95% CI 0.84‐1.09).

The stricter outcomes sensitivity analyses conducted showed that although 96.2% of participants reported at least one respiratory symptom, 75.8% reported two or more respiratory symptoms, and 56.5% met the clinically focused respiratory composite of shortness of breath with persistent cough and/or chest discomfort. The mean respiratory symptom count was 2.31 symptoms (SD 1.05).

In adjusted models, no covariate was significantly associated with two or more respiratory symptoms, the clinically focused respiratory composite or respiratory symptom count. Outdoor occupational exposure showed positive but non‐significant associations with two or more respiratory symptoms (aOR 1.35, 95% CI 0.75‐2.43; *p* = 0.317) and the clinical respiratory composite (aOR 1.42, 95% CI 0.85‐2.37; *p* = 0.182). In the Poisson model, outdoor occupational exposure was also not associated with respiratory symptom count (IRR 1.05, 95% CI 0.89‐1.24; *p* = 0.587). Smoking, pre‐existing respiratory disease, older age, sex and mask use were not consistently associated with stricter respiratory‐health outcomes. Overall, respiratory burden remained high using more restrictive definitions, but no stable independent predictors of higher respiratory burden were identified within this health‐seeking outpatient sample.

#### 3.2.3 Alternative Model

In an alternative multivariable model incorporating more granular exposure proxies, including occupational exposure category and ranked daily outdoor hours, patterns were broadly consistent with those observed in the primary analysis. Compared with indoor workers, participants classified as having mixed occupational exposure did not show significantly different odds of reporting respiratory symptoms (aOR = 0.74, 95% CI 0.16‐3.42), whereas those in the high outdoor exposure group had higher odds of symptom reporting, although this association was not statistically significant and was accompanied by wide confidence intervals (aOR = 2.13, 95% CI 0.47‐9.43). Increasing duration of daily outdoor exposure, modelled as an ordinal variable, was not independently associated with respiratory symptoms (aOR = 0.81, 95% CI 0.31‐1.91).

Current smoking emerged as a significant predictor in this model, with smokers exhibiting higher odds of respiratory symptom reporting compared with non‐smokers (aOR = 3.49, 95% CI 1.06‐13.35). Pre‐existing respiratory disease and older age were not independently associated with symptom reporting after adjustment. Mask use was associated with lower odds of respiratory symptoms (aOR = 0.31, 95% CI 0.09‐1.07), suggesting a potential protective effect, although this association narrowly failed to reach statistical significance. As in the primary model, sex was strongly associated with symptom reporting, with male participants having substantially lower odds of reporting respiratory symptoms compared with female participants (aOR = 0.05, 95% CI 0.00‐0.31; *p* = 0.007). This alternative modelling approach, which explicitly accounted for occupational exposure category and duration of outdoor activity, did not materially alter the direction of associations observed in the primary analysis.

### 3.3 Behavioural Protection Analysis

In the behavioural protection analysis assessing whether mask use modified the association between outdoor occupational exposure and respiratory symptom reporting, no evidence of effect modification was observed. In the fully adjusted logistic regression model, neither outdoor occupational exposure nor mask use alone was independently associated with respiratory symptoms. Outdoor work was associated with lower odds of symptom reporting, although this association was not statistically significant (aOR = 0.55, 95% CI 0.12‐2.33). Mask use was similarly associated with lower odds of respiratory symptoms (aOR = 0.53, 95% CI 0.10‐4.00); however, this estimate was imprecise and did not reach statistical significance.

The interaction term between outdoor work and mask use was not statistically significant (aOR = 0.93, 95% CI 0.09‐8.38; *p* = 0.95). Current smoking status and pre‐existing respiratory disease were included as covariates but were not independently associated with respiratory symptom reporting in this model, and increasing age was likewise not associated with the outcome. Although overall model fit improved modestly relative to the null model, the absence of a statistically significant interaction suggests that mask use did not measurably modify the relationship between outdoor exposure and respiratory symptoms in this cross‐sectional analysis. Assessment of multicollinearity in the behavioural protection model indicated no evidence of problematic collinearity among predictors. Variance inflation factor values for the main effects ranged from 1.13 to 2.78, whereas the interaction term exhibited a higher VIF value (3.53), as expected for higher order terms. All values remained below commonly accepted thresholds.

#### 3.3.1 Predicted Probabilities

**FIGURE 4:**
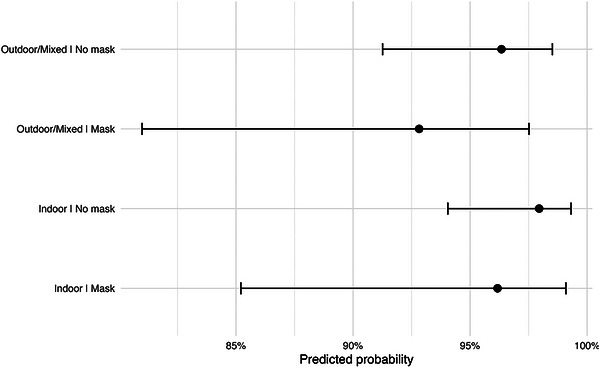
Predicted probability of any respiratory symptom by outdoor exposure and mask use.

Predicted probabilities derived from the behavioural protection model were used to aid interpretation of the interaction between outdoor occupational exposure and mask use (Figure 4). After holding smoking status, pre‐existing respiratory disease and age at their sample means, the estimated probability of reporting any respiratory symptom was high across all exposure and behaviour groups. Among indoor workers who did not report mask use, the predicted probability of respiratory symptoms was 0.98 (95% CI 0.94‐0.99), compared with 0.96 (95% CI 0.85‐0.99) among indoor workers who reported mask use. For participants with outdoor or mixed occupational exposure, the predicted probability was 0.96 (95% CI 0.91‐0.99) among those not using masks and 0.93 (95% CI 0.81‐0.98) among those reporting mask use. Although predicted probabilities were consistently lower among participants who reported mask use across both indoor and outdoor exposure groups, the differences between groups were modest and were accompanied by overlapping confidence intervals.

### 3.4 Symptom Burden Profiling

In the symptom burden profiling analysis, overall symptom count was modelled using Poisson regression, as no evidence of overdispersion was detected. After adjustment for key exposure and behavioural variables, outdoor occupational exposure was not significantly associated with total symptom burden (IRR = 1.07, 95% CI 0.96‐1.19). Similarly, current smoking status was not independently associated with symptom count (IRR = 1.09, 95% CI 0.98‐1.22), and mask use showed no association with overall symptom burden (IRR = 1.00, 95% CI 0.89‐1.12).

To further characterise heterogeneity in symptom presentation, participants were grouped according to symptom patterns using an unsupervised clustering approach. Three distinct symptom clusters were identified. The largest cluster was characterised by relatively low overall symptom burden (mean symptom count of 3.0) and low non‐respiratory symptom prevalence and was classified as the low‐burden group. A second cluster exhibited elevated levels of both respiratory and non‐respiratory symptoms, with a mean symptom count of 3.6 and comparable contributions from respiratory and non‐respiratory complaints and was labelled the mixed multisystem group. The third cluster demonstrated the highest symptom burden, with a mean symptom count of 4.4 and a predominance of respiratory symptoms and was classified as the respiratory‐dominant group.

Multinomial logistic regression analyses were subsequently conducted to examine whether occupational exposure, smoking status or mask use predicted membership in higher symptom‐burden clusters, with the low‐burden cluster as the reference category. None of the examined variables were significantly associated with membership in either the respiratory‐dominant or mixed multisystem clusters. Outdoor occupational exposure was not associated with increased relative risk of belonging to the respiratory‐dominant cluster (RRR = 0.87, 95% CI 0.51‐1.46) or the mixed multisystem cluster (RRR = 0.96, 95% CI 0.60‐1.53). Similarly, current smoking was not significantly associated with cluster membership for either the respiratory‐dominant cluster (RRR = 1.26, 95% CI 0.73‐2.17) or the mixed multisystem cluster (RRR = 1.30, 95% CI 0.80‐2.12). Mask use was associated with lower relative risk of membership in both higher burden clusters; however, these associations did not reach statistical significance for either the respiratory‐dominant cluster (RRR = 0.72, 95% CI 0.41‐1.28) or the mixed multisystem cluster (RRR = 0.78, 95% CI 0.47‐1.30).

### 3.5 Ambient Air Quality and Pollutant Trends During the Smog Season

**FIGURE 5:**
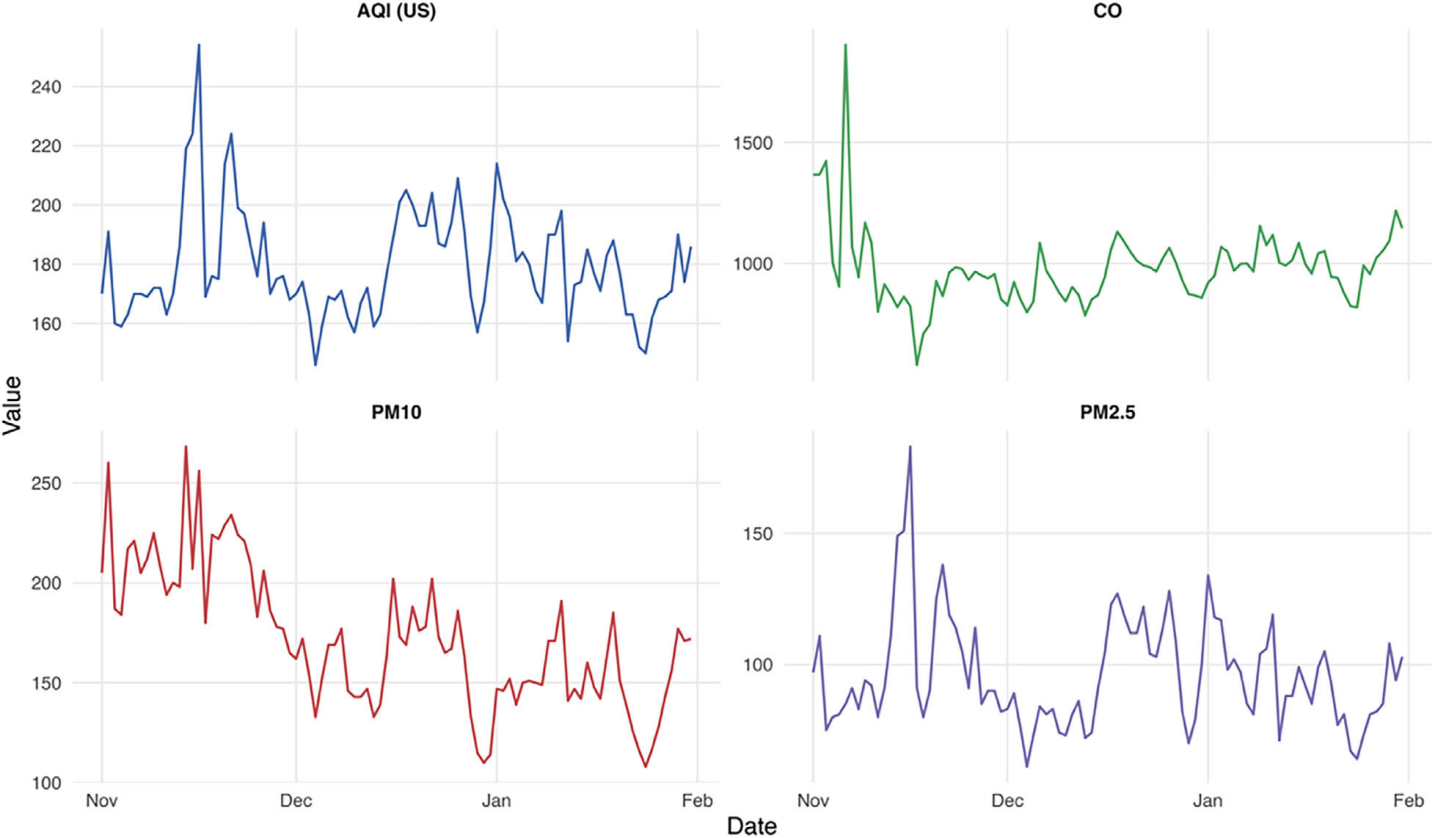
Daily air quality indicators (contextual environmental data). AQI, air quality index; CO, carbon monoxide.

Time‐series analysis of ambient air quality indicators demonstrated persistently poor air quality throughout the study period, spanning November 2024‐January 2025 (Figure 5). The AQI remained consistently unhealthy, with repeated peaks and limited periods of improvement. Monthly summaries corroborated these visual trends. Mean AQI values remained consistently high across all 3 months, with monthly means of 184 in November, 178 in December and 178 in January, and maximum values reaching as high as 254. When aggregated across the whole study period, the overall mean AQI was 180, with a 95th percentile value of 214 and a maximum of 254, indicating frequent and substantial departures from acceptable air quality levels.

Particulate matter concentrations were similarly elevated and exhibited marked temporal variability. PM_10_ concentrations were highest during the early smog period, with a mean of 210 in November, before declining modestly in December and January, though remaining consistently elevated throughout. Over the full study period, PM_10_ had an overall mean concentration of 172, a 95th percentile of 227 and a maximum of 268. PM_2.5_ concentrations followed a comparable pattern, with particularly high levels observed in November (mean 102), followed by persistently elevated concentrations in December and January. PM_2.5_ exhibited a mean concentration of 96.2, a 95th percentile of 131, and peak values reaching 183, indicating sustained exposure to fine particulate matter during the smog season. CO concentrations also remained elevated throughout the study period, with substantial short‐term variability. Monthly mean CO concentrations ranged from 938 to 1010, with particularly high maximum values observed in November. Across the study period, the mean CO concentration was 979, with a 95th percentile of 1191 and a maximum of 1903, reflecting persistent combustion‐related pollution during the smog season.

### 3.6 Perceived Air Quality

#### 3.6.1 Perceived Air Quality and Vulnerability Indicators

Perception‐related findings were interpreted cautiously because recruitment was restricted to outpatients who presented with perceived worsening health during the smog season. As a result, the perception variable was likely affected by selection bias and should not be generalised as a measure of air quality awareness or risk perception among Lahore residents.

Exploratory analyses examined whether perceived worsening air quality differed across vulnerability and exposure‐related characteristics. Cross‐tabulation and chi‐square testing showed no significant association between perceived deteriorating air quality and outdoor occupational exposure or pre‐existing respiratory disease. Perception of worsening air quality differed by smoking status, although this finding should be interpreted cautiously given the selected health‐seeking sample and the possibility of sparse data.

To further explore predictors of perceived worsening air quality, multivariable logistic regression was conducted. Because of quasi‐separation in the outcome distribution, Firth penalised logistic regression was used to obtain more stable estimates than conventional logistic regression. In this exploratory model, current smoking was associated with higher odds of perceived worsening air quality; however, the estimate was extremely imprecise, with a very wide confidence interval, suggesting sparse data or residual separation rather than a stable independent effect (aOR = 131.38, 95% CI 17.32‐16,896.55; *p* < 0.001). Male sex was associated with lower odds of perceiving worsening air quality relative to female sex (aOR = 0.01, 95% CI 0.00‐0.05; *p* < 0.001), although this finding should also be interpreted in the context of recruitment‐related selection and potential differences in symptom reporting or healthcare‐seeking behaviour.

Outdoor occupational exposure was not independently associated with perceived worsening air quality (aOR = 1.24, 95% CI 0.46‐3.19; *p* = 0.663) nor was pre‐existing respiratory disease (aOR = 0.50, 95% CI 0.21–1.16; *p* = 0.108). Age was not associated with perceived worsening air quality (aOR = 0.98 per year, 95% CI 0.94‐1.02; *p* = 0.295), and mask use showed no independent association with perceived air quality (aOR = 1.02, 95% CI 0.29‐3.87; *p* = 0.977). Estimates from conventional logistic regression were unstable and yielded implausibly wide confidence intervals, reinforcing that these perception‐related analyses should be regarded as exploratory and hypothesis‐generating.

#### 3.6.2 Perceived Air Quality and Symptom Reporting

Additional analyses examined whether perceived worsening air quality was associated with respiratory symptom reporting and overall symptom burden after adjustment for exposure proxies and individual characteristics. These analyses were also interpreted cautiously because perceived worsening health during the smog season formed part of the recruitment context, which may have reduced the independence of perception‐related variables.

In the multivariable logistic regression model examining the presence of any respiratory symptom, perceived worsening air quality was not independently associated with respiratory symptom reporting (aOR = 1.25, 95% CI 0.24–5.40). Outdoor occupational exposure similarly showed no independent association with respiratory symptoms (aOR = 1.43, 95% CI 0.40‐4.68). Smoking status was associated with higher odds of respiratory symptoms, although this association was imprecise and did not reach statistical significance (aOR = 2.89, 95% CI 0.77‐11.95). Pre‐existing respiratory disease and age were also not independently associated with respiratory symptom reporting. Male sex remained associated with lower odds of reporting respiratory symptoms than female sex (aOR = 0.05, 95% CI 0.00‐0.37). Mask use was associated with lower odds of respiratory symptoms, but the confidence interval included the null value (aOR = 0.30, 95% CI 0.09‐1.06).

In the Poisson model for total symptom count, perceived worsening air quality was not associated with overall symptom burden (IRR = 0.94, 95% CI 0.76–1.18). Outdoor occupational exposure was likewise not associated with symptom count (IRR = 1.06, 95% CI 0.93‐1.21). Current smoking was associated with higher symptom burden (IRR = 1.15, 95% CI 1.00‐1.32), whereas pre‐existing respiratory disease, age, sex and mask use were not independently associated with symptom count.

### 3.7 Contextual Ambient Air Quality and Temperature Conditions During the Smog Season

**FIGURE 6:**
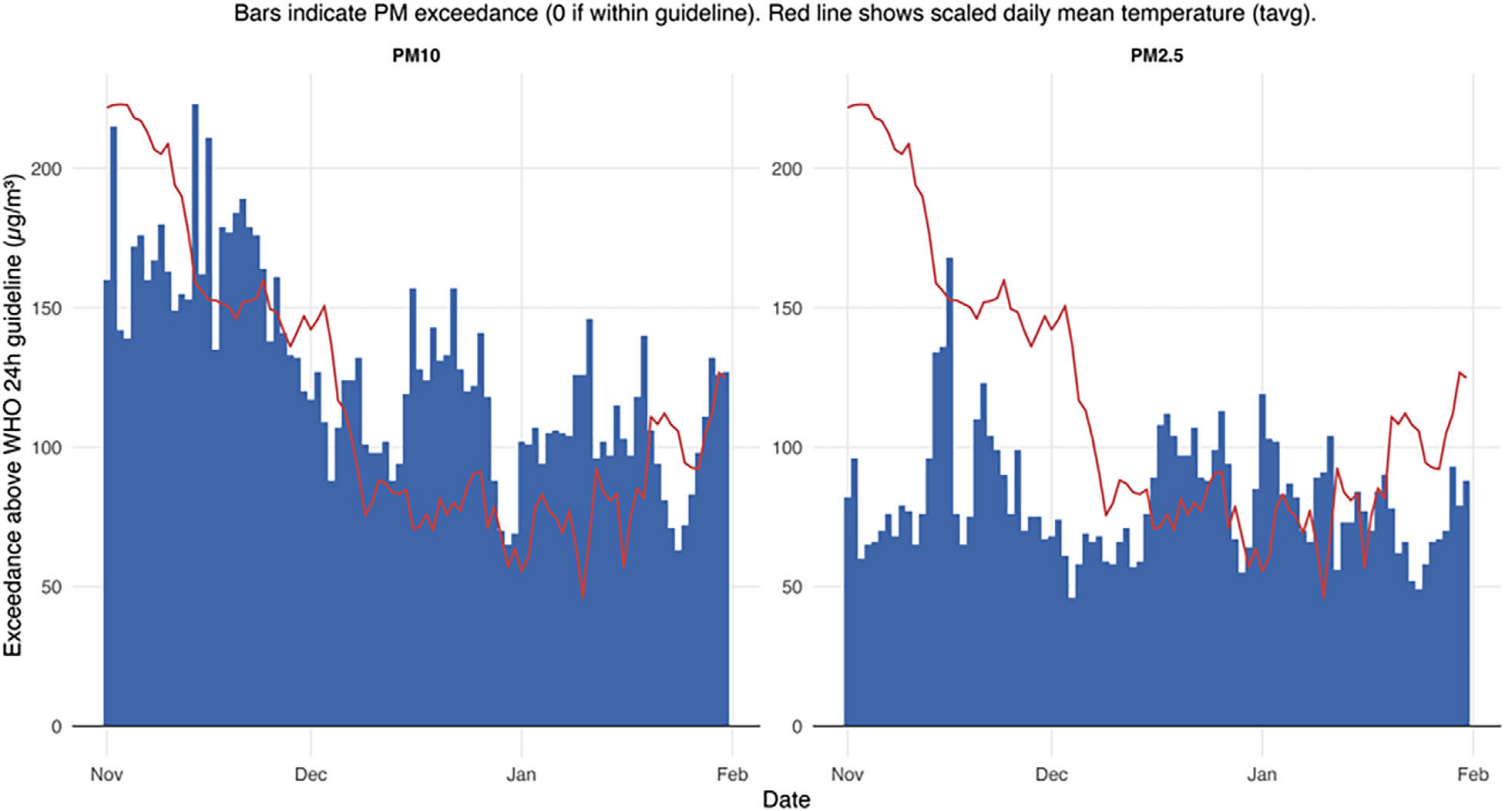
Daily PM exceedance above WHO guidelines with ambient temperature context.

Contextual analysis of ambient environmental data demonstrated sustained and severe air quality degradation throughout the study period, spanning November 2024–January 2025, alongside cool seasonal temperatures (Figure 6). Daily monitoring data indicated persistently elevated concentrations of particulate matter and CO throughout the 92‐day period. Across the study period, PM_2.5_ exhibited a mean concentration of 96.2, a median of 91, a 95th percentile of 131 and peak levels of 183. PM_10_ concentrations were similarly elevated, with an overall mean of 172, a median of 170 and a 95th percentile of 227, with maximum values up to 268. CO concentrations remained consistently high, with a mean of 979, a 95th percentile of 1191 and peak concentrations reaching 1903. Comparison with the WHO 24‐h air quality guidelines revealed pervasive exceedance during the study period. Both PM_2.5_ and PM_10_ concentrations exceeded guideline thresholds on 100% of monitored days, indicating continuous exposure to particulate matter levels well above recommended limits. Temperature data further contextualised these conditions. Mean daily ambient temperature during the study period was 14.8°C, with a 95th percentile of 23.0°C. Mean daily minimum and maximum temperatures were 9.3°C and 21.7°C, respectively, with maximum daily temperatures reaching up to 29.6°C. These relatively cool conditions are consistent with wintertime atmospheric stagnation, which may contribute to pollutant accumulation and reduced dispersion.

### 3.8 Persistence and Intensity of PM_2.5_ Guideline Exceedance During the Smog Season

**FIGURE 7:**
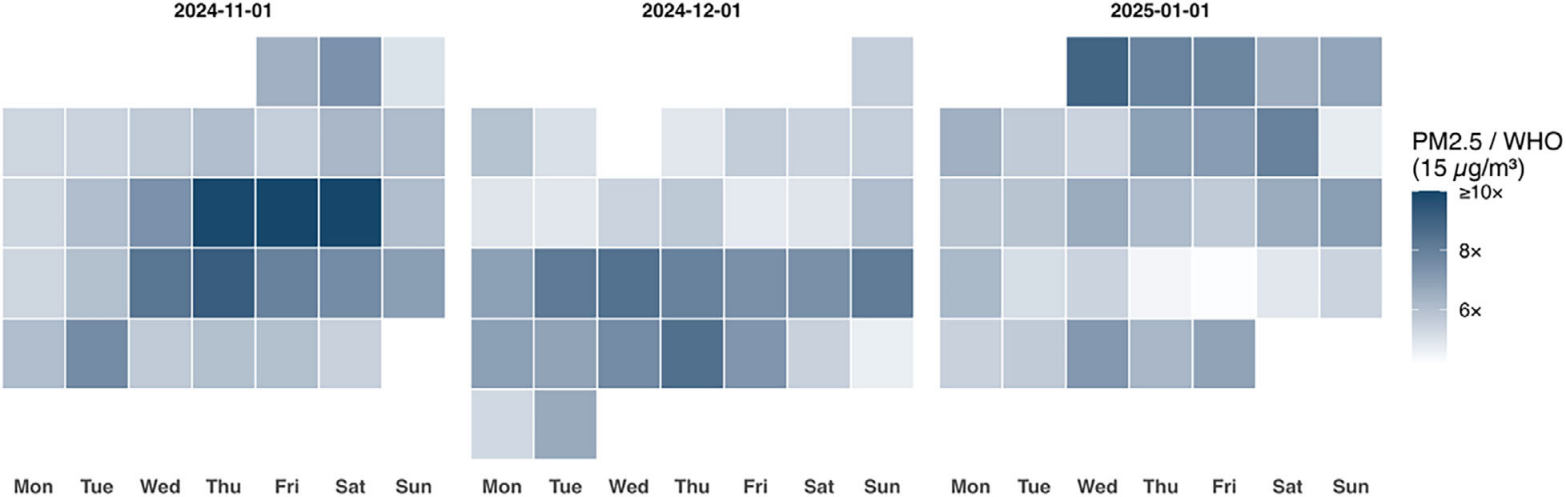
Daily exceedance of the WHO PM_2.5_ guideline during the Lahore smog season.

The calendar heatmap illustrates sustained and severe exceedance of the WHO 24‐h PM_2.5_ guideline (15 µg/m^3^) throughout the Lahore smog season from November 2024 to January 2025 (Figure 7). Across all 3 months, exceedances were frequent and often substantial, with many days recording PM_2.5_ concentrations several times the recommended limit. The intensity of exceedance is conveyed by darker shading, indicating days when PM_2.5_ levels reached 8‐ to 10‐fold or more the WHO guideline.

In November 2024, prolonged clusters of high‐intensity exceedance were evident, particularly during mid‐ to late‐month periods, suggesting persistent exposure rather than isolated pollution episodes. December 2024 showed continued widespread exceedance, with fewer low‐intensity days and several weeks characterised by consistently elevated PM_2.5_ levels. In January 2025, although some days exhibited comparatively lower exceedance intensity, guideline breaches remained common.

## 4 Discussion

In settings where ambient air pollution is extreme and persistent, individual‐level exposure contrasts may diminish, limiting the utility of conventional exposure‐response approaches. In this outpatient sample, severe city‐level ambient pollution coincided with a high burden of reported symptoms, underscoring the importance of interpreting the findings within the monitored environmental context. Against this backdrop, we observed an alarmingly high symptom burden during Lahore's winter smog season, consistent with monitoring‐informed evidence from other heavily polluted regions. Nearly all participants reported at least one acute symptom, with respiratory complaints (shortness of breath, cough and sore throat) especially prevalent. This aligns with prior research from Delhi and Lahore, where severe smog episodes have been associated with widespread respiratory and ocular symptoms [11, 12]. In our cohort, eye irritation affected over half of participants, and non‐respiratory complaints such as fatigue and headaches were common, mirroring patterns reported during Delhi's 2024 pollution crisis as monitored air quality deteriorated [11]. These convergent findings suggest that extreme particulate pollution, documented through routine environmental monitoring, corresponds with a broad spectrum of health complaints extending beyond the respiratory system. Although our analysis centred on human health, the same atmospheric conditions captured by urban monitoring networks that drive severe smog are likely to affect urban ecosystems, livestock and wildlife, reinforcing the need to interpret air quality data within an integrated environmental, ecological and public health framework rather than solely as a human health issue.

The cumulative vulnerability of individuals emerged as a key correlate of symptom burden in our study. Participants with multiple risk factors, such as pre‐existing respiratory disease, smoking, older age and high‐exposure occupations, experienced higher total symptom counts than less vulnerable individuals. This dose‐response pattern is consistent with evidence that pollution disproportionately affects those with underlying susceptibilities. For instance, an analysis in Lahore found that individuals with chronic respiratory conditions had approximately four‐fold higher odds of coughing and six‐fold higher odds of breathlessness during smog periods [12]. Similarly, studies in China have identified smokers and those with respiratory comorbidities as sub‐populations who report more acute symptoms under heavy haze conditions [13]. Our results reinforce these trends; current smokers in our cohort had a modest but significantly higher symptom count than non‐smokers, and those with prior respiratory illness tended to report more symptoms (though confidence intervals were wide). This suggests that smog exposure amplifies health burdens most in people who already carry vulnerabilities, in line with global understanding of air pollution risk profiles [14, 15, 16].

The sensitivity analyses using stricter respiratory‐health outcomes further support this interpretation. The original ‘any respiratory symptom’ endpoint was extremely common and had limited discriminatory value; however, even when respiratory burden was defined as two or more symptoms, a clinically focused respiratory composite, or respiratory symptom count, no stable independent predictors of higher respiratory burden were identified. This suggests that the absence of strong associations in the primary model was not solely an artefact of using an overly broad binary outcome. Rather, within this health‐seeking outpatient sample, respiratory symptoms were widespread across vulnerability and occupational categories, consistent with the possibility that severe city‐wide ambient pollution reduced meaningful contrast between broad exposure groups.

Occupational exposure gradients were less influential in our adjusted analyses than initially expected. We observed a higher crude prevalence of certain symptoms (shortness of breath, cough, sore throat and eye irritation) among the high outdoor‐exposure group than among indoor workers. However, after accounting for other factors, working outdoors was not a significant independent predictor of symptom reporting. One interpretation is that when pollution is ubiquitous and severe, as it was in Lahore, where 100% of days during the study exceeded WHO 24‐h safety guidelines for PM_2.5_ and PM_10_ by substantial margins, even ‘indoor’ individuals are still continuously exposed to unhealthy air [17, 18, 19]. In such conditions, the distinction between indoor and outdoor exposure narrows; ambient pollutants likely infiltrate homes and workplaces, and most people must spend some time outside for daily activities. A comparable cross‐sectional study in the Lahore region similarly found that people in both outdoor and indoor occupations were ‘equally affected’ by winter smog, with no statistically significant difference in overall symptom rates between these groups [12]. Thus, our findings align with regional data suggesting that in extremely polluted urban environments, ambient smog exposure is effectively universal [20, 21], potentially overwhelming any protective effect of staying indoors without specialised air filtration.

Our data also did not demonstrate a clear protective benefit of routine mask use against symptoms, nor any significant interaction between mask‐wearing and outdoor work. Participants who reported wearing face masks tended to have slightly lower predicted probabilities of respiratory symptoms, but this trend was modest and not statistically significant. It is important to interpret this cautiously; the lack of an observed effect does not prove masks are ineffective but rather may reflect limitations such as inconsistent mask quality or usage, and the study's cross‐sectional design. Many participants may have used basic cloth or surgical masks, which offer only partial filtration for fine particles. In real‐world conditions of extreme smog (where daily PM_2.5_ levels often reached eight to ten times the WHO guideline), even diligent mask wearers could experience high cumulative exposure. Our results suggest that individual‐level precautions, such as standard face masks or staying indoors, provided only limited mitigation during Lahore's smog crisis, a finding consistent with public health experience that only stringent pollution control and filtration (far beyond what most individuals can implement) truly reduce exposure during such severe events [22, 23, 24].

An unexpected finding was a significant sex difference in symptom reporting: Female participants had substantially higher odds of reporting respiratory symptoms than male participants, even after adjusting for exposure and health factors. This result mirrors observations in the environmental health literature that women may experience greater health impacts from air pollution, potentially due to physiological differences or activity patterns [25, 26]. However, this contrasts with other reports in the literature in which gender was not a significant predictor of pollution‐related health effects, as meta‐analyses have found no consistent sex/gender differences in short‐term associations between air pollution and cardiovascular mortality [27]. In our context, the sex disparity might be influenced by healthcare‐seeking behaviour (women attending the clinic could have been more symptomatic or health‐conscious) or unmeasured factors such as indoor cooking smoke exposure that disproportionately affects women in South Asia [28]. Further investigation would be needed to determine whether this reflects a true biological differential susceptibility or a sociocultural artefact in reporting. Regardless, the result underscores the importance of considering gender in vulnerability assessments for pollution health effects.

Another important aspect of our study is the discordance between subjective air quality perception and health status. We found that a large share of participants who did not perceive the air as ‘worsening’ during the smog season nevertheless reported a high symptom burden. Conversely, some who believed air quality had deteriorated reported few symptoms. This misalignment suggests that personal awareness of pollution is an imperfect indicator of actual health risk. Many individuals may habituate to chronic air pollution and fail to recognise incremental worsening, even as their bodies experience ill effects. Others might attribute symptoms (e.g., cough or fatigue) to factors such as seasonal illness or stress rather than pollution. Prior research indicates that risk perception can indeed diverge from objective exposure; for example, during China's 2013 ‘Airpocalypse’, public concern spiked only when pollution reached outrageous levels, even though health harm was accumulating continuously [29]. Similarly, a study in Bangladesh found that people's self‐reported health status related to air pollution did correlate with perceived pollution, but the relationship was complex and mediated by factors like education and awareness [30]. Our findings reinforce that a lack of perceived pollution does not equate to safety; many participants reported symptoms despite not perceiving worsening air quality. This has public health implications: Authorities cannot rely solely on public complaints or perceptions to gauge smog's impact, and risk communication must acknowledge that even ‘invisible’ or normalised pollution levels can be harmful.

In the broad context, the health impacts observed in Lahore are consonant with the well‐documented consequences of severe air pollution worldwide. The situation in Lahore during late 2024, with a maximum daily AQI of 254 in the city‐level dataset analysed here and PM_2.5_ concentrations several times above WHO limits, was extreme by current global standards. Only historic disasters, like the Great London Smog of 1952, or more recent wildfire crises, approach this level of sustained pollution intensity. Those episodes likewise led to abrupt spikes in respiratory and cardiovascular morbidity and mortality. For example, emergency room visits for respiratory causes surged significantly during China's 2013 haze disaster and during acute wildfire smoke events in North America [31]. During the 2023 Canadian wildfire smoke events that affected large parts of the contiguous United States, asthma‐related emergency department visits were approximately 17% higher than expected on days with wildfire smoke, highlighting that even short‐term exposures to wildfire fine particulate matter can provoke substantial respiratory health responses [32]. Compared to these episodic crises, Lahore's smog season represents a protracted public health emergency. The cumulative toll of living under hazardous air for months on end is likely far greater, encompassing not just acute symptom burden as captured in our study, but also longer term risks of cardiopulmonary diseases, stroke and other outcomes documented in epidemiological studies. Air pollution is a leading environmental cause of premature death globally, with the World Health Organisation estimating approximately 7 million deaths annually due to combined ambient and household air pollution, and with South Asia shouldering a disproportionately large share of this burden [21, 33]. Our on‐the‐ground data from Lahore add a human dimension to these statistics, illustrating how pollution translates into day‐to‐day suffering: difficulty breathing, irritated eyes, disturbed sleep, anxiety and general ill health among the outpatients studied. These patterns suggest that smog operates as a social‐ecological stressor that disproportionately burdens individuals already facing health or socioeconomic vulnerability, reinforcing existing urban health inequities.

Many high‐income regions have achieved significant improvements in air quality over the past decades, offering a hopeful contrast. Stricter regulations and emission controls in Europe and North America have eliminated mainly the kind of persistent smog that once plagued cities like London, Los Angeles and Pittsburgh. Average PM_2.5_ levels in those cities today are a fraction of Lahore's winter values, and routine exceedances of WHO guidelines are now infrequent (although they still occur), and the WHO has tightened standards to reflect health effects at even low levels [34, 35, 36]. The experiences of those regions show that air pollution mitigation can yield tangible health benefits, from lower respiratory illness rates to improved life expectancy. However, the recent spikes in Western cities from wildfires and heat‐related stagnation events also demonstrate that vigilance is needed even in cleaner environments, as climate change and other factors can abruptly worsen air quality. Ultimately, comparing Lahore's situation with global contexts underscores both the universality of air pollution's health risks and the importance of policy action: Where pollution has been reduced, health has improved; conversely, where extreme pollution persists, communities continue to endure preventable health burdens.

Although this study provides insight into smog‐related health effects in an underrepresented setting, several limitations should be considered when interpreting the findings. The high prevalence of symptoms should be interpreted in light of the recruitment strategy. Because participants were enrolled from outpatient attendees who presented with perceived worsening health during the smog season, the study likely overestimates symptom prevalence relative to the general Lahore population. Health outcomes were self‐reported, which introduces the possibility of reporting and recall bias, as participants may have under‐ or over‐estimated symptoms based on personal perception or concurrent illnesses. The absence of clinical validation or objective health measurements means that some misclassification of symptoms is likely, particularly for common winter complaints that may not be directly attributable to air pollution.

The outpatient sample was recruited from a single tertiary care hospital, which may limit generalisability to the broader Lahore population. Individuals seeking medical care during the smog season are likely to have a higher underlying symptom burden than the general community, and certain groups may be underrepresented due to healthcare access or sociocultural factors. As a result, absolute symptom prevalence estimates should be interpreted cautiously, although internal comparisons between exposure and vulnerability groups remain informative.

Individual‐level air pollution exposure was not directly measured, and exposure assessment relied on occupational and behavioural proxies, along with city‐level ambient air quality data. This approach may have led to exposure misclassification, as substantial variability in personal exposure can occur within the same urban environment. The lack of temporal linkage between symptom onset and daily pollution levels further limits inference about short‐term exposure–response relationships and may have attenuated observed associations.

The cross‐sectional design precludes causal inference and limits assessment of symptom progression or long‐term health impacts. Residual confounding from unmeasured factors, such as indoor air pollution sources, socioeconomic status, seasonal infections or psychosocial stress, cannot be excluded. These limitations suggest that the results should be interpreted as descriptive and hypothesis‐generating, highlighting patterns of health burden during the smog season rather than establishing definitive causal relationships.

Despite these limitations, the study provides real‐world evidence from a high‐pollution setting that has been underrepresented in the literature. By integrating symptomatic data with environmental context, our work contributes to understanding the symptom burden reported during periods of extreme smog. The results, while exploratory, are consistent with prior studies, lending them validity even as we acknowledge the constraints. Future research can build on this by employing more robust exposure measurements, expanding to additional participant groups (including healthy community members and multiple cities) and exploring interventions (such as air purifiers or public health advisories) to mitigate the health effects of South Asia's recurring smog seasons. Our findings underscore an urgent need for policy action: Reducing emissions and improving air quality in Lahore and similar megacities is critical to alleviate the preventable suffering associated with seasonal smog, a conclusion that resonates with experiences from across the globe.

## 5 Conclusion

This study documents substantial respiratory and non‐respiratory symptom burden among adult outpatients attending a single tertiary hospital during Lahore's 2024–2025 winter smog season. Symptoms were widespread across occupational categories, whereas city‐level monitoring showed persistently severe ambient pollution throughout the study period. Higher exploratory vulnerability scores were associated with greater symptom burden, whereas individual protective behaviours showed limited measurable benefit. PM_2.5_ and PM_10_ exceeded World Health Organization guideline values throughout the monitoring period, providing environmental context for the observed symptom patterns. These observations support integrated emissions control, land‐use planning, air quality management and public health responses rather than reliance on individual‐level mitigation alone.

## Author Contributions

**Mirza Ameer Faizan Ali**: conceptualization, methodology, investigation, data curation. **Bushra Iftikhar**: investigation, resources, data curation. **Syed Zeeshan Haider**: supervision, project administration. **Aqsa Ikram**: methodology. **Ayesha Masood**: investigation, resources. **Ghaniya Ali**: investigation, resources. **Jude Oluwapelumi Alao**: formal analysis, visualisation, writing – original draft. **Eunice Damilola Wilkie**: writing – original draft, writing – review and editing.

## Funding

The authors have nothing to report.

## Ethical Statement

Ethical approval was obtained from the Institutional Review Board of Al Aleem Medical College, Lahore (Approval No. EA‐52‐2024). Written informed consent was obtained from all participants before enrolment. All data were anonymised, securely stored and analysed in aggregate to protect participant confidentiality.

## Consent

The authors have nothing to report.

## Conflicts of Interest

The authors declare no conflicts of interest.

## Supporting information




**Supporting File:** puh270349‐sup‐0001‐SuppMat.docx.

## Data Availability

The anonymised participant data, environmental monitoring data, data dictionary, and analysis code supporting the findings of this study are publicly available in the Zenodo repository at https://doi.org/10.5281/zenodo.21694004. All data necessary to reproduce the analyses reported in this article are included in the repository and supplementary File 1.

## References

[1] Health Effects Institute , State of Global Air 2025 (Health Effects Institute, 2025), https://www.healthdata.org/sites/default/files/2025‐10/soga‐2025‐report.pdf.

[2] Copernicus , CAMS Global System Tracks Exceptional Air Pollution Episode in South Asia (European Commission, 2024), https://atmosphere.copernicus.eu/cams‐global‐system‐tracks‐exceptional‐air‐pollution‐episode‐south‐asia.

[3] H. Irfan , “Air Pollution and Cardiovascular Health in South Asia: A Comprehensive Review,” Current Problems in Cardiology 49, no. 2 (2024): 102199, 10.1016/j.cpcardiol.2023.102199. 37977414

[4] F. Khanum , M. N. Chaudhry , and P. Kumar , “Characterization of Five‐Year Observation Data of Fine Particulate Matter in the Metropolitan Area of Lahore,” Air Quality, Atmosphere and Health 10, no. 6 (2017): 725–736, 10.1007/s11869-017-0464-1. PMC5581825 28936270

[5] H. Regan and S. Saifi , Toxic Smog in Pakistan Is so Bad You Can See It From Space (CNN, 2024), https://www.cnn.com/2024/11/11/asia/pakistan‐punjab‐pollution‐satellite‐images‐climate‐intl‐hnk.

[6] S. Khan , “From Bad to Worse: The Unbreathable Air of Lahore,” Lancet 404, no. 10471 (2024): 2537–2538, 10.1016/S0140-6736(24)02601-1. 39709198

[7] berkeleyearth.org. (n.d.). Accessed 1/02/2026.

[8] V. C. Pun , F. Kazemiparkouhi , J. Manjourides , and H. H. Suh , “Long‐Term PM_2.5_ Exposure and Respiratory, Cancer, and Cardiovascular Mortality in Older US Adults,” American Journal of Epidemiology 186, no. 8 (2017): 961–969, 10.1093/aje/kwx166. 28541385 PMC6915823

[9] WHO , WHO Global Air Quality Guidelines: Particulate Matter (PM_2.5_ and PM_10_), Ozone, Nitrogen Dioxide, Sulfur Dioxide and Carbon Monoxide, 1st ed. (World Health Organization, 2021). 34662007

[10] D. Saadi , E. Tirosh , and I. Schnell , “The Relationship Between City Size and Carbon Monoxide (CO) Concentration and Their Effect on Heart Rate Variability (HRV),” International Journal of Environmental Research and Public Health 18, no. 2 (2021): 788, 10.3390/ijerph18020788. 33477714 PMC7831902

[11] A. Bhuyan , T. Bordoloi , R. Debnath , A. M. A. Ikbal , B. Debnath , and W. S. Singh , “Assessing AQI of Air Pollution Crisis 2024 in Delhi: Its Health Risks and Nationwide Impact,” Discover Atmosphere 3, no. 1 (2025): 13, 10.1007/s44292-025-00041-x.

[12] F. Jabeen , Z. Ali , and A. Maharjan , “Assessing Health Impacts of Winter Smog in Lahore for Exposed Occupational Groups,” Atmosphere 12, no. 11 (2021): 1532, 10.3390/atmos12111532.

[13] X. Yang , L. Liu , Y. Li , et al., “Differences in Perceived Sensitivity to Air Pollution Between Smokers and Non‐Smokers During a Heavy Haze Episode in Northeast China,” Scientific Reports 15, no. 1 (2025): 26476, 10.1038/s41598-025-12248-4. 40691318 PMC12280120

[14] M. L. Bell , A. Zanobetti , and F. Dominici , “Who Is More Affected by Ozone Pollution? A Systematic Review and Meta‐Analysis,” American Journal of Epidemiology 180, no. 1 (2014): 15–28, 10.1093/aje/kwu115. 24872350 PMC4070938

[15] L. G. Hooper and J. D. Kaufman , “Ambient Air Pollution and Clinical Implications for Susceptible Populations,” Annals ATS 15, no. S2 (2018): S64–S68, 10.1513/AnnalsATS.201707-574MG. PMC5955035 29676646

[16] P. Zhao , C. Xie , S. Wang , et al., “The Causal Relationship Between Long‐Term Exposure to Major PM_2.5_ Constituents and the Rate of Emergency Department Visits: A Difference‐in‐Differences Study,” Toxics 13, no. 11 (2025): 973, 10.3390/toxics13110973. 41304525 PMC12656412

[17] Y. Hu , J. S. Ji , and B. Zhao , “Deaths Attributable to Indoor PM_2.5_ in Urban China When Outdoor Air Meets 2021 WHO Air Quality Guidelines,” Environmental Science & Technology 56, no. 22 (2022): 15882–15891, 10.1021/acs.est.2c03715. 36278921

[18] S. J. Pai , T. S. Carter , C. L. Heald , and J. H. Kroll , “Updated World Health Organization Air Quality Guidelines Highlight the Importance of Non‐Anthropogenic PM_2.5_ ,” Environmental Science & Technology Letters 9, no. 6 (2022): 501–506, 10.1021/acs.estlett.2c00203. 35719860 PMC9202349

[19] J. Rentschler and N. Leonova , “Global Air Pollution Exposure and Poverty,” Nature Communications 14, no. 1 (2023): 4432, 10.1038/s41467-023-39797-4. PMC10363163 37481598

[20] M. N. Azimi and M. M. Rahman , “Unveiling the Health Consequences of Air Pollution in the World's Most Polluted Nations,” Scientific Reports 14, no. 1 (2024): 9856, 10.1038/s41598-024-60786-0. 38684837 PMC11058277

[21] World Health Organization , Ambient (Outdoor) Air Pollution (World Health Organization, 2024), https://www.who.int/news‐room/fact‐sheets/detail/ambient‐(outdoor)‐air‐quality‐and‐health.

[22] F. T. Lu , R. J. Laumbach , A. Legard , et al., “Real‐World Effectiveness of Portable Air Cleaners in Reducing Home Particulate Matter Concentrations,” Aerosol and Air Quality Research 24, no. 1 (2024): 230202, 10.4209/aaqr.230202. 38618024 PMC11014421

[23] M. M. Maestas , R. D. Brook , R. A. Ziemba , et al., “Reduction of Personal PM_2.5_ Exposure via Indoor Air Filtration Systems in Detroit: An Intervention Study,” Journal of Exposure Science and Environmental Epidemiology 29, no. 4 (2019): 484–490, 10.1038/s41370-018-0085-2. 30420725 PMC7021209

[24] M. Thompson , R. Castorina , W. Chen , D. Moore , K. Peerless , and S. Hurley , “Effectiveness of Air Filtration in Reducing PM_2.5_ Exposures at a School in a Community Heavily Impacted by Air Pollution,” Atmosphere 15, no. 8 (2024): 901, 10.3390/atmos15080901.

[25] J. E. Clougherty , “A Growing Role for Gender Analysis in Air Pollution Epidemiology,” Environmental Health Perspectives 118, no. 2 (2010): 167–176, 10.1289/ehp.0900994. 20123621 PMC2831913

[26] G. Liu , B. Sun , L. Yu , et al., “The Gender‐Based Differences in Vulnerability to Ambient Air Pollution and Cerebrovascular Disease Mortality: Evidences Based on 26781 Deaths,” Global Heart 15, no. 1 (2020): 46, 10.5334/gh.849. 32923340 PMC7427691

[27] U. Kraus , S. Horstmann , L. Dandolo , G. Bolte , A. Peters , and A. Schneider , “Sex/Gender in the Association Between Ambient Air Pollution and Cardiovascular Mortality: Systematic Review and Meta‐Analysis,” Ecotoxicology and Environmental Safety 300 (2025): 118443, 10.1016/j.ecoenv.2025.118443. 40466332

[28] K. Dash , M. R. Behera , H. M. Degge , S. Nayak , D. Behera , and J. R. Mohanty , “Insights Into Household Air Pollution: A Systematic Scoping Review on Perceptions of Solid Biomass Fuel Use in South Asian Countries,” Journal of Family Medicine and Primary Care 14, no. 8 (2025): 3159–3178, 10.4103/jfmpc.jfmpc_1817_24. PMC12488177 41041209

[29] J. M. Ferreri , R. D. Peng , M. L. Bell , L. Ya , T. Li , and G. Brooke Anderson , “The January 2013 Beijing “Airpocalypse” and Its Acute Effects on Emergency and Outpatient Visits at a Beijing Hospital,” Air Quality, Atmosphere and Health 11, no. 3 (2018): 301–309, 10.1007/s11869-017-0538-0. PMC6918940 31853329

[30] M. M. Rahman , A. B. M. Hasanuzzaman , M. A. Chisty , E. Alam , M. K. Islam , and A. Islam , “Perceived‐Air Pollution and Self‐Reported Health Status: A Study on Air Pollution‐Prone Urban Area of Bangladesh,” Frontiers in Public Health 13 (2025): 1382471, 10.3389/fpubh.2025.1382471. 40247869 PMC12003290

[31] F. Liang , L. Tian , Q. Guo , et al., “Associations of PM2.5 and Black Carbon With Hospital Emergency Room Visits During Heavy Haze Events: A Case Study in Beijing, China,” International Journal of Environmental Research and Public Health 14, no. 7 (2017): 725, 10.3390/ijerph14070725. 28678202 PMC5551163

[32] C. E. McArdle , T. C. Dowling , K. Carey , et al., “Asthma‐Associated Emergency Department Visits During the Canadian Wildfire Smoke Episodes—United States, April–August 2023,” MMWR Morbidity and Mortality Weekly Report 72, no. 34 (2023): 926–932, 10.15585/mmwr.mm7234a5. 37616233 PMC10468220

[33] Health Effects Institute , Database of South Asia–Air Pollution and Health (DoSAAH) (Health Effects Institute, 2024), https://www.healtheffects.org/announcements/database‐south‐asia‐air‐pollution‐and‐health‐dosaah.

[34] European Environment Agency , Air Quality Status Report 2025 (Publications Office, 2025), https://data.europa.eu/doi/10.2800/9895153.

[35] Greater London Authority , London Atmospheric Emissions Inventory LAEI 2022 (Greater London Authority, 2022), https://data.london.gov.uk/dataset/london‐atmospheric‐emissions‐inventory‐laei‐2022‐2lg5g.

[36] U.S. Environmental Protection Agency , Final Regulatory Impact Analysis for the Reconsideration of the National Ambient Air Quality Standards for Particulate Matter (U.S. Environmental Protection Agency, 2024).

