# Supplementary material for: Health Under Haze: Symptom Burden Among Adult Outpatients During the 2024‐2025 Winter Smog Season in Lahore, Pakistan
Source: Public Health Chall. 2026 Aug 17;5(3):e70349. doi: 10.1002/puh2.70349 (PMC13481319; doi:10.1002/puh2.70349)
Supplement: Supplementary file 1 [file PUH2-5-e70349-s001.docx]

**QUESTIONNAIRE**

**Effect of Smog on the health of patients attending Out-Patient Department of Gulab Devi Teaching Hospital, Lahore**

**Demographical Investigation:**

Age: _______________ Gender­­­­­: _____________Occupation: ______________________

Smoking Status: ____________ Residential Area: ________________­

**Do you have any pre-existing health conditions?**

- Asthma
- Chronic Obstructive Pulmonary Disease (COPD)
- Cardiovascular Disease
- Diabetes
- None
- Other (Specify: ____________)

**How often are you outdoors during smoggy conditions per day?**

1) Less than 1 hour	**2)** 1–3 hours/day

**3)** >3 hours/day	**4) >**5 hours/day

**Do you use protective measures (masks, or any other)?**

1) Yes	**2)** No

If yes (Specify: ____________)

**Have you noticed a worsening of air quality in your area recently?**

**1)** Yes	**2)** No

**Do you commute during high AQI times (early morning, evening rush hours)?**

**1)** Yes	**2)** No

**Have you experienced any of the following symptoms in the past month?**

- Persistent cough
- Shortness of breath
- Eye irritation
- Sore throat
- Fatigue
- Chest pain
- Skin irritation
- Other (Specify: ____________)

**If yes, when did these symptoms start?**

- Less than 1 week ago
- 1–4 weeks ago
- More than 4 weeks ago

**Have you visited a doctor for these symptoms recently?**

**1)** Yes	**2)** No

**Were you diagnosed with a smog-related health condition?**

**1)** Yes	**2)** No

If yes (Specify: ____________)

**Have your daily activities been affected by smog exposure?**

**1)** Yes	**2)** No

**If yes, in what way?**

- Reduced physical activity
- Increased fatigue
- Avoiding outdoor tasks
- Other (Specify: ____________)

**Have you experienced frequent infections during smoggy periods?**

**1)** Yes	**2)** No

If yes (Specify: ____________)

**Have you undergone any medical tests for infections during smog season?**

**1)** Yes	**2)** No

If yes (Specify: ____________)

**Are you aware of the Air Quality Index (AQI) and its health implications?**

**1)** Yes	**2)** No

**Do you believe smog has impacted your health?**

**1)** Yes	**2)** No

**Have you felt stressed or anxious about the smog and its health effects?**

**1)** Yes	**2)** No

**How would you rate your stress level during smoggy conditions?**

- 1 (No stress)
- 2 (Mild stress)
- 3 (Moderate stress)
- 4 (High stress)
- 5 (Extreme stress)

**Have you experienced any of the following during smoggy periods?**

- Sleep disturbances
- Palpitation
- Reduced productivity at work/school
- Difficulty concentrating
- Loss of appetite
- Feelings of depression or hopelessness
- Other (Specify: ____________)

**Have you reduced social activities or gatherings due to smog?**

**1)** Yes	**2)** No

**Do you think smog has led to increased conflicts or tension at home or work?**

**1)** Yes	**2)** No

**Financial burdens caused by government-imposed restrictions addressing smog.**

1. Yes	**2)** No
